# Promoting female physician-scientists: Perspectives from a unique learning environment

**DOI:** 10.1017/cts.2023.38

**Published:** 2023-04-04

**Authors:** Ashti M. Shah, Rashmi J. Rao

**Affiliations:** Physician Scientist Training Program, University of Pittsburgh School of Medicine, Pittsburgh, PA 15213, USA

**Keywords:** Physician-scientist, women, gender-gap, medical-education, graduate-education

Despite progress toward gender equity in academic medicine over the years, female physician-scientists represent only 20% of editors-in-chief at top-ranked medical journals and are less likely to be grant-awardees or in positions of leadership [1–3]. As aspiring female physician-scientists, we have found ourselves in a unique environment that gave us insight into overcoming the gender gap in academic medicine. At the University of Pittsburgh School of Medicine’s Physician Scientist Training Program (PSTP), a selective 5-year longitudinal research training program for medical students [4], 58% of students, both past and present, have been female. Through applicant demographics and matriculation serendipity, our PSTP class of 11 students is 81% female. As we sit in a female-majority classroom, we asked the question: what empowers us to pursue a career in academic medicine, despite the challenges we may face as women in the field? We believe that our female-majority classroom inadvertently highlighted strategies for cultivating a well-rounded mentor network and fostered professional development skills to advance our careers, all while allowing us to remain authentic to ourselves.

While gender concordance between mentor and mentee does not correlate with mentorship satisfaction [5], we took this opportunity to explore whether gender concordance between mentor and mentee helps increase our self-confidence that we too could become successful physician-scientists. Data indicated that the majority of students in the PSTP over the years, both male (42%) and female (58%), chose male research mentors (66%). However, women chose female mentors more than men (31% vs. 21%), suggesting women may prefer female mentors, but the availability of these (R01-funded, primarily MD or MD-PhD) mentors is the limiting factor. This highlights the need for the PSTP to increase the number of female physician-scientist mentors that students are introduced to, formally through classes or otherwise.

To address this limitation, our program encourages students to develop a diverse set of mentors including academic, clinical, personal, and lifestyle mentors whose range of expertise help guide us through the demands of academia, work, and life. While a single mentor may not perfectly fit our needs, curating a set of mentors who guide us in different domains gives us the structural support and confidence that we can be successful female physician-scientists. For example, the data shown above demonstrate that most students choose to work with a male PI for their research year, most likely due to the alignment of research interests between the student and PI. Yet, many female students have identified female physician-scientist mentors, through our PSTP career advisor program or informally, to discuss topics such as career and family planning, workplace dynamics, and work-life balance. The introduction to multiple mentors allows students in the PSTP to circumvent the present gap in high-achieving female mentors.

Besides the lack of female mentors, another challenge faced by women, is that they are perceived as significantly less ambitious and driven than men, which may account for the lack of women in the highest ranks of academic medicine [6]. Paradoxically, self-promoting women who showcase their ambition and drive have decreased social acceptance [7]. In the PSTP, we have learned to comfortably self-promote our accomplishments in competitive environments with help from executive coaches. The PSTP provides each student with a professional career consultant (with an MD or PhD), who is an expert in facilitating strategic planning and decisions aligned with personal goals. Interestingly, over 60% of students in our class discussed the topic of confidence with their career coach. The career consultants advised us to promote each other’s recent achievements in group settings, thus giving way for self-promotion while maintaining social acceptance.

This exercise has found a unique place in practicing our elevator-pitches. In addition to preparing our own pitch, we also practice giving our peers’ elevator-pitch to the rest of the class. This teaches us how to promote our own work, while encouraging us to promote each other’s work in group settings. We have extended these practices to lab meetings, clinical rotations, and conferences. Collaborative self-promotion limits competition and allows us to maintain our social acceptance, while also advancing our careers.

While 3 of 6 PSTP enrichment courses are joint with Medical Scientist Training Program, MDPhD students (54% female), our experiences suggest that specific elements of the PSTP classroom allow for the rise in self-efficacy in female trainees (reported by 90% of female PSTP students). Preliminary data from biannual surveys evaluating measures of grit, motivation, confidence, and satisfaction show that women express lower level of confidence in their ability to execute professional or scientific tasks at baseline, but that their confidence levels increase faster than those of men. Our unique PSTP cohort allowed us to answer: what does our learning environment offer that allows for the extent of female empowerment we have experienced? We are convinced that fostering relationships with a diverse set of mentors and exercises to increase self- and peer promotion are central to creating a level playing field in academic.

## References

[1] Hart KL , Perlis RH . Trends in proportion of women as authors of medical journal articles, 2008–2018. JAMA Internal Medicine 2019; 179(9): 1285–1287. DOI: 10.1001/jamainternmed.2019.0907. 31135815 PMC6547141

[2] Pinho-Gomes A-C , Vassallo A , Thompson K , Womersley K , Norton R , Woodward M . Representation of women among editors in chief of leading medical journals. JAMA Network Open 2021; 4(9): e2123026–e2123026. DOI: 10.1001/jamanetworkopen.2021.23026. 34495341 PMC8427369

[3] Lewiss RE , Silver JK , Bernstein CA , Mills AM , Overholser B , Spector ND . Is academic medicine making mid-career women physicians invisible? Journal of Women’s Health 2020; 29(2): 187–192. 10.1089/jwh.2019.7732 31593525

[4] Steinman RA , Proulx CN , Levine AS . The highly structured Physician Scientist Training Program (PSTP) for medical students at the University of Pittsburgh. Academic Medicine 2020; 95(9): 1373. 32079926 10.1097/ACM.0000000000003197 PMC7447180

[5] Farkas AH , Bonifacino E , Turner R , Tilstra SA , Corbelli JA . Mentorship of women in academic medicine: a systematic review. Journal of General Internal Medicine 2019; 34(7): 1322–1329. DOI: 10.1007/s11606-019-04955-2. 31037545 PMC6614283

[6] Eagly AH , Nater C , Miller DI , Kaufmann M , Sczesny S . Gender stereotypes have changed: A cross-temporal meta-analysis of US public opinion polls from 1946 to 2018. American Psychologist 2020; 75(3): 301. 31318237 10.1037/amp0000494

[7] Rudman LA . Self-promotion as a risk factor for women: the costs and benefits of counterstereotypical impression management. Journal of Personality and Social Psychology 1998; 74(3): 629. 9523410 10.1037//0022-3514.74.3.629

